# DNA methylation microarrays identify epigenetically regulated lipid related genes in obese patients with hypercholesterolemia

**DOI:** 10.1186/s10020-020-00220-z

**Published:** 2020-10-07

**Authors:** Teresa Płatek, Anna Polus, Joanna Góralska, Urszula Raźny, Anna Gruca, Beata Kieć-Wilk, Piotr Zabielski, Maria Kapusta, Krystyna Słowińska-Solnica, Bogdan Solnica, Małgorzata Malczewska-Malec, Aldona Dembińska-Kieć

**Affiliations:** 1 Department of Clinical Biochemistry, Jagiellonian University Medical College, Kopernika 15a, 31-501 Kraków, Poland; 2 Department of Metabolic Diseases, Jagiellonian University Medical College, Kopernika 15a, 31-501 Kraków, Poland; 3 Department of Metabolic Diseases, University Hospital in Krakow, Jakubowskiego 2, 30-688 Kraków, Poland; 4 Department of Physiology, Medical University of Bialystok, Mickiewicza 2C, 15-222 Białystok, Poland

**Keywords:** DNA methylation, Obesity, Hypercholesterolemia, Plasma lipids

## Abstract

**Background:**

Epigenetics can contribute to lipid disorders in obesity. The DNA methylation pattern can be the cause or consequence of high blood lipids. The aim of the study was to investigate the DNA methylation profile in peripheral leukocytes associated with elevated LDL-cholesterol level in overweight and obese individuals.

**Methods:**

To identify the differentially methylated genes, genome-wide DNA methylation microarray analysis was performed in leukocytes of obese individuals with high LDL-cholesterol (LDL-CH, ≥ 3.4 mmol/L) versus control obese individuals with LDL-CH, < 3.4 mmol/L. Biochemical tests such as serum glucose, total cholesterol, HDL cholesterol, triglycerides, insulin, leptin, adiponectin, FGF19, FGF21, GIP and total plasma fatty acids content have been determined. Oral glucose and lipid tolerance tests were also performed. Human DNA Methylation Microarray (from Agilent Technologies) containing 27,627 probes for CpG islands was used for screening of DNA methylation status in 10 selected samples. Unpaired t-test and Mann–Whitney U-test were used for biochemical and anthropometric parameters statistics. For microarrays analysis, fold of change was calculated comparing hypercholesterolemic vs control group. The q-value threshold was calculated using moderated Student’s t-test followed by Benjamini–Hochberg multiple test correction FDR.

**Results:**

In this preliminary study we identified 190 lipid related CpG loci differentially methylated in hypercholesterolemic versus control individuals. Analysis of DNA methylation profiles revealed several loci engaged in plasma lipoprotein formation and metabolism, cholesterol efflux and reverse transport, triglycerides degradation and fatty acids transport and β-oxidation. Hypermethylation of CpG loci located in promoters of genes regulating cholesterol metabolism: *PCSK9, LRP1, ABCG1, ANGPTL4, SREBF1* and *NR1H2* in hypercholesterolemic patients has been found*.* Novel epigenetically regulated CpG sites include *ABCG4, ANGPTL4, AP2A2, AP2M1, AP2S1, CLTC, FGF19, FGF1R, HDLBP, LIPA, LMF1, LRP5, LSR, NR1H2* and *ZDHHC8* genes*.*

**Conclusions:**

Our results indicate that obese individuals with hypercholesterolemia present specific DNA methylation profile in genes related to lipids transport and metabolism. Detailed knowledge of epigenetic regulation of genes, important for lipid disorders in obesity, underlies the possibility to influence target genes by changing diet and lifestyle, as DNA methylation is reversible and depends on environmental factors. These findings give rise for further studies on factors that targets methylation of revealed genes.

## Background

The worldwide prevalence of obesity nearly tripled between 1975 and 2016 and is still growing, contributing to an increased incidence of comorbidities such as type 2 diabetes, dyslipidemia, liver steatosis, hypertension, cardiovascular disease (CVD) and cancer (Blüher 2019). Metabolic complications of obesity include insulin resistance, impaired secretion and action of incretin hormones, disturbed plasma lipoprotein clearance and metabolism and low grade inflammation (Pedersen 2013; Chia and Egan 2020; Magkos et al. 2008; Ellulu et al. 2016). A number of factors can play a role in weight gain. Among them, the most powerful factors seem to be an unhealthy lifestyle and genetics (Albuquerque et al. 2017). Research over the last decade indicates that various environmental factors at different stages of life can changes to chromatin structure and function and thus change cellular phenotype and metabolism (Rosen et al. 2018; Piening et al. 2018; Jacobsen et al. 2012; Stuart et al. 2018; Roh et al. 2018). Epigenetic modifications such as DNA methylation and multiple histone post-translational modifications regulate gene transcription and thus adapt metabolism to environmental factors (Handel et al. 2010; Keating and El-Osta 2015). It is estimated that 70% of promoters in human genomes are rich in CpG sites indicating that DNA methylation serves as a crucial epigenetic modification (Blattler and Farnham 2013). DNA methylation of CpG sites in the genes’ promoters as well as distal regulatory sites may modify gene expression by altering the interaction of histones, thereby affecting the binding of transcription factors or recruitment of methyl-CpG binding proteins (MBPs) (Rottach et al. 2009; Handel et al. 2010). Methylation of CpG dinucleotides may be reversible, modified in response to environmental factors consequently repressing or activating transcription (Blattler and Farnham 2013; Handel et al. 2010; Abdul et al. 2017). Current scientific reports show that methylation of CpG islands influences the expression of genes related to obesity, metabolic syndrome and type 2 diabetes (Kim et al. 2015; Ali et al. 2016; Shen and Zhu 2018; Guo et al. 2020). We hypothesize that several processes regulating lipid levels are controlled by DNA methylation. Therefore, this study aimed to investigate the differences in DNA methylation status in leukocytes of obese subjects with hypercholesterolemia compared to controls. Here we present the results of an analysis of a genome-wide methylation profile, with focus on genes involved in lipids metabolism to find the pathways mostly affected by hypercholesterolemia and find new candidates genes. The results of this work may contribute to a better understanding of the epigenetic mechanisms related to dyslipidemia.

## Methods

### Aim of the study

The study aims to elucidate the link between epigenetic changes and hypercholesterolemia in obese patients.

### Patients

Our cohort involved 137 individuals with BMI (body mass index) above normal range (min.27–max.45 kg/m^2^), women (n = 99) and men (n = 38), aged 25 to 65 years. Exclusion criteria included cardiovascular diseases, diabetes mellitus, kidney or liver failure, endocrine disorders, chronic inflammation, hormone therapy, use of lipid-lowering or anti-inflammatory drugs, use of diet supplements, smoking or excessive use of alcohol, pregnancy or lactation.

The hypercholesterolemia group, (n = 68) consisted of patients with obesity with borderline high, or high serum LDL cholesterol (LDL-CH) levels (≥ 3.4 mmol/L) and the control group (n = 69) consisted of obese subjects with serum LDL-CH levels < 3.4 mmol /L (Stone et al. 2013). At this cut-off point the control group included patients with optimal for low CV risk [≤ 3.0 mmol/L according to recent ESC/EAS 2019 guidelines (Mach et al. 2019)] as well as near-optimal LDL-CH concentration (3.0–3.4 mmol/L). The main criteria of enrolment into study groups were: overweight or obesity without comorbidities requiring treatment (except hypertension treated with AT1 receptor antagonists or calcium channel blockers) and fasting LDL-CH ≥ 3.4 mmol/L (group with newly diagnosed hypercholesterolemia) or LDL-CH < 3.4 mmol/L (control group).

From the cohort 10 samples representative for both groups were selected for the study of DNA methylation on microarrays.

### Anthropometric parameters and blood pressure

Anthropometric parameters: body weight, height, waist and hip circumferences were measured and BMI, waist to hip ratio (WHR) were calculated. Body fat percentage was estimated by bioelectrical impedance method using the Segmental Body Composition Analyzer TANITA BC 418 MA. Blood pressure was measured after 10 min of rest.

### Sample collection

Fasting venous blood samples were collected and centrifuged (1000×*g* for 10 min at 4 °C within 30 min from collection) for serum and plasma separation. Serum and plasma samples were immediately frozen and stored at − 80 °C for further analyses of glucose, insulin, adipokines (leptin and adiponectin), glucose-dependent insulinotropic peptide (GIP), fibroblast growth factor 19 (FGF19), fibroblast growth factor 21 (FGF21), total cholesterol, HDL (high density lipoprotein)-cholesterol, triglycerides (TGs), total plasma fatty acids content and composition. For analysis of DNA methylation fasting peripheral blood was collected into K_3_-EDTA-containing tubes and stored at − 80 °C until analysed.

Oral glucose tolerance test (OGTT) and oral lipid tolerance test (OLTT) were performed on separate days. Venous blood samples: fasting, 30, 60, 90 and 120 min of OGTT as well as fasting (before meal), 2, 4, 6, and 8 h of OLTT were collected in order to measure postprandial glucose, insulin, GIP and TG serum concentrations. OLTT- an 8-h high fat mixed meal tolerance test that contained 73% fat, 16% protein, and 11% carbohydrates, with a caloric value of 1018 kcal was performed. The detailed composition of meal was described previously (Razny et al. 2018).

### Biochemical tests

Serum glucose, total cholesterol, HDL cholesterol, and TGs were measured using enzymatic colorimetric methods on the MaxMat analyzer (MaxMat S.A., Montpeliere, France). LDL cholesterol concentration was calculated using the Friedewald formula. Serum insulin was determined by immunoradiometric method (Diasource, ImmunoAssays, Belgium). Serum leptin, adiponectin, FGF19 and FGF21 were measured using ELISA (Human Leptin Quantikine ELISA kit; Human Total Adiponectin/Acrp30 Quantikine ELISA kit; Human FGF-19 Quantikine ELISA Kit; Human FGF-21 Quantikine ELISA Kit, respectively, R&D Systems Inc. Minneapolis, MN, USA). GIP was measured by ELISA [Human GIP (Total) ELISA kit (EMD Millipore, St Charles, MO, USA)]. Total plasma fatty acids content and composition was measured by gas–liquid chromatography and flame-ionization detector after direct in situ transesterification, according to Glaser et al. (2010). Plasma fatty acids profile included quantitative determination of saturated (myristic, palmitic, stearic, behenic, lignoceric, and arachidic), monounsaturated (palmitoleic, oleic and nervonic) and polyunsaturated (arachidonic, linoleic, α-linolenic, eicosapentaenoic, and docosahexaenoic) fatty acids.

### DNA methylation screening analysis

To perform DNA methylation screening, taking into account that both groups of hypercholesterolemia and control were equal-sized, five samples for microarrays analyses were randomly selected from each of them. To reduce the difference between structure of participants in two original groups and their random subsamples, randomization was carried out in sex stratas in proportions of women to men 3:2 to map numerical superiority of men in our study (99 women versus 38 men). We didn’t consider any other sampling strata given the limited sample size of a participants on which we could measure genome wide methylation. Drawn individuals in hypercholesterolemic and control groups were balanced by age and BMI.

### Methylation screening analysis with immunoprecipitation of methylated DNA and hybridization to Human DNA Methylation Microarray

Genomic DNA from venous blood was extracted using the High Pure PCR Template Preparation Kit (ROCHE Diagnostics, Mannheim, Germany). The measurement of DNA quantity and quality was performed by spectrophotometry using the NanoDrop ND1000. An amount of 5 μg of DNA was taken for sonication. Sonication efficiency was assessed by electrophoresis on a 2.0% agarose gel. The sonicated DNA sample was then divided into two aliquots: four parts of DNA were taken for immunoprecipitation, the fifth part was stored as a reference input fraction. Analysis of methylated DNA was done by immunoprecipitation of DNA containing 5-methylcytosines (5-mC) using monoclonal antibodies against 5-methylcytidine (Monoclonal Antibody to 5-Methyl Cytosine/5-MeC Purified from Acris Antibodies, Inc, USA). Immunoprecipitated and reference samples were labelled with fluorescent dyes Cyanine-3 and Cyanine-5, respectively. The exact steps were performed based on the methodology of Agilent Technologies. Competitive hybridization of input material and methylated enriched DNA was performed to oligonucleotide microarrays—Human DNA Methylation Microarray (G4495A, Design ID, 023795) from Agilent Technologies. High-definition 244 K arrays contained 27,627 probes for annotated human CpG islands and 5081 for Undermethylated Regions (UMRs). Microarrays were hybridized for 40 h at 65 °C. Slides washing and scanning procedures and image extraction using Agilent Features Extraction software v 10.10.1.1 were performed according to the manufacturer instructions.

### Statistical analyses

For biochemical and anthropometric parameters, Shapiro–Wilk test was used to assess normality of distribution of continuous variables, then unpaired t-test for normally distributed data and Mann–Whitney U-test for non-normally distributed data were used for comparison of the two groups. The Chi-squared test was used for nominal variables. Normally distributed data are shown as mean ± standard deviation (SD), otherwise as median (Q2) and interquartile range in parentheses (Q1; Q3). All analyses were performed with the Statistica 13 software (StatSoft). The p-value < 0.05 was considered statistically significant.

Microarray data analysis was performed using the Feature extraction software v 10.10.1.1 (Agilent Technologies, Santa Clara, USA), the BRB-ArrayTools Version 4.6 software (National Institutes of Health, Bethesda, MD, USA), R programming language (R Foundation for Statistical Computing, University of Auckland, New Zealand) and the Gene Spring version 13 software (Agilent Technologies, Santa Clara, USA). Feature extraction software was used to assess background subtracted intensity values for the two fluorescence dyes on each individual array feature and calculated as the ratio (Cy3/Cy5). We used Lowess normalization method for dual channel raw hybridization signals and background correction to median of all samples. A quality analysis was performed for each sample array taken for analysis (QC reports evaluation). Regarding methylation sites we removed bad quality probes, probes not located in CpG islands, probes containing SNPs in the CpG site and removed probes located on X and Y chromosomes. To account for potential differences in the proportions of blood cells, we estimated the proportion of Lymphocytes, Monocytes, Eosinophils, Basophils and Neutrophils to adjust raw data as previously described by Houseman et al. in R (Houseman et al. 2014). Fold of change was calculated for the hypercholesterolemic group in relation to the control group and shown as methylation level. The p-value threshold was calculated using statistical filtering (moderated Student’s t-test followed by Benjamini–Hochberg multiple test correction FDR-q-value). Loci corresponding to a q-value of < 0.05 and fold change of either > 1.3 or < − 1.3 were classified as differentially methylated. Highly methylated regions had ratios significantly above zero while less methylated regions had log ratios significantly below zero.

Pathways analysis was performed in Reactome Pathway to obtained the list of differentially methylated genes related to lipids pathways (Sidiropoulos et al. 2017). Subsequently we used the BiNGO plugin in Cytoscape software (version 3.7.2) to assessed the involvement of selected genes in biological processes and molecular function (Maere et al. 2005). We used parameters as: overrepresentation after correction (using statistical test as hypergeometric test with multiple testing correction as Benjamini and Hochberg False Discovery Rate (FDR) correction). Results with corrected p-value < 0.05 are presented in manuscript.

## Results

### Biochemical and anthropometric characteristics of groups

The studied group of patients with high serum LDL-CH was comparable to the control group in terms of weight, BMI, WHR, blood pressure and adipose tissue mass. The hypercholesterolemic group showed not only higher LDL-CH but also total cholesterol, triglycerides, non-HDL-CH compared to the control group (Table 1). Total plasma fatty acids content and saturated fatty acids content were higher in the high LDL-CH group as well. Particularly, higher content of palmitoleic acid (mean 1.33 (µg/mL) ± 0.18 vs mean 0.84 (µg/mL) ± 0.34, p = 0.0352) was observed in hypercholesterolemic group. Additionally, higher percentage of myristic acid (mean 0.87 (%) ± 0.79 vs mean 0.35 (%) ± 0.15, p = 0.188) in this group was noted.

**Table 1:** Characteristics of subjects selected for DNA methylation analysis

	Hypercholesterolemia group (n = 5)	Control group (n = 5)	p-value
Age (years)	43.0 ± 12.6	44.6 ± 10.6	0.83
Sex, female (%)^b^	60	60	ns
Weight (kg)^a^	88 (80–107.7)	89.2 (80.2–103.2)	1
Adipose tissue mass (%)^a^	38.3 (30.55–43.25)	37.4 (30.9–45.3)	0.47
BMI (kg/m^2^)	34.0 (29.47–36.9)	31.7 (28.4–38.9)	0.676
WHR^a^	0.9 (0.81–0.95)	0.81 (0.79–0.82)	0.11
Systolic blood pressure (mmHg)^a^	130 (127.5–140)	124 (119–130)	0.095
Diastolic blood pressure (mmHg)^a^	80 (80–90)	80 (77–86)	0.53
Total cholesterol (mmol/L)^a^	6.31 (5.58–6.64)	5.0 (4.5–5.25)	0.06
*LDL cholesterol (mmol/L)*^a^	*4.27 (3.9–4.8)*	*2.74 (2.5–2.95)*	*< 0.001*
HDL cholesterol (mmol/l)^a^	1.2 ± 0.3	1.4 ± 0.3	0.25
*Non-HDL (mmol/L)*^a^	*5.19 (4.53–5.37)*	*3.49 (2.99–4.11)*	*0.02*
Fasting triglycerides (mmol/L)	1.7 ± 0.7	1.1 ± 0.8	0.26
Total fatty acids (µg/mL)	4118 ± 1226	3341 ± 925	0.31
Saturated fatty acids (%)	34.04 ± 1.96	32.09 ± 0.85	0.08
Monounsaturated fatty acids (%)	28.52 ± 4.35	26.42 ± 4.42	0.49
Polyunsaturated fatty acids (%)	37.43 ± 6.02	41.49 ± 4.37	0.28
Fasting glucose (mmol/L)^a^	5.8 (5.1–6.3)	4.9 (4.7–5.8)	0.4
*Glucose AUC OLTT (mmol/L min)*^a^	*10.44 (10.0–10.72)*	*9.05 (8.78–9.77)*	*0.022*
Glucose AUC OGTT (mmol/L min)^a^	4.4 (3.2–5.0)	3.26 (2.6–3.45)	0.06
Fasting insulin (µIU/mL)	22.8 ± 16.8	11.3 ± 4.2	0.173
HOMA-IR	4.0 ± 1.6	2.6 ± 1.1	0.14
Adiponectin (µg/mL)^a^	4.2 (3.55–8.07)	7.4 (5.58–10.0)	0.21
Leptin (ng/mL)^a^	29.89 (15.18–46.04)	21.8 (14.16–101.85)	0.83
Fasting GIP (pg/mL)^a^	32.54 (12.3–48.8)	24.1 (18.1–37.43)	0.4
FGF19 (pg/mL)	160.8 ± 61.7	151.4 ± 108.7	0.62
FGF21 (pg/mL)	280.4 ± 144.3	138.8 ± 92.5	0.102

Comparison between the high LDL-CH group and controls (unpaired t-test for normally or Mann–Whitney U-test for non-normally distributed ^a^ variables). Data shown as mean ± SD; otherwise (^a^) as median and interquartile range in parentheses except nominal variable (^b^) shown as % of subjects. ^b^ The chi-squared test was used for nominal variable. The statistically significant results are marked in italics

Fasting serum TG levels were higher in the hypercholesterolemic patients. Obese subjects had fasting glucose below the upper reference limit, though slightly higher values of serum glucose were observed in the dyslipidemic patients compared to control. Subjects with high plasma LDL-CH were also characterized by higher FGF21 and lower adiponectin (presenting trend toward significance) serum levels (Table 1).

As the group studied in terms of methylation was a representative part of the larger cohort, below we present the characteristics of the entire cohort in Table 2. The trends and directions of differences between microarray subgroups have been preserved for those parameters that differed significantly in large groups, especially the proportions of fatty acid groups and the concentrations of GIP, FGF19 and FGF21 proteins.

**Table 2:** Characteristics of subjects with obesity included into the study, comparison of groups according to fasting serum LDL cholesterol

	Hypercholesterolemic group (n = 68)	Control group (n = 69)	p-value
*Age (years)*	*50.1 ± 9.8*	*44.6 ± 12.2*	*< 0.004*
Sex, female (%)^b^	75	70	ns
Weight (kg)^a^	92.2 (79.87–104.5)	92.5 (84–104.2)	0.425
Adipose tissue mass (%)^a^	39.8 (34.4–42.8)	38.3 (33.2–42.2)	0.24
BMI (kg/m^2^)^a^	33.0 (30.6–35.6)	32.1 (30.1–35.7)	0.45
WHR^a^	0.9 (0.83–0.98)	0.85 (0.8–0.975)	0.23
Systolic blood pressure (mmHg)^a^	130 (120–140)	126 (120–135)	0.16
Diastolic blood pressure (mmHg)^a^	85 (80–90)	81 (80–89.5)	0.14
*Total cholesterol (mmol/L)*^a^	*6.14 (5.57–6.75)*	*4.88 (4.42–5.1)*	*< 0.001*
*LDL cholesterol (mmol/L)*^a^	*4.14 (3.82–4.57)*	*2.91 (2.5–3.2)*	*< 0.001*
HDL cholesterol (mmol/L)	1.3 ± 0.3	1.3 ± 0.3	0.69
*Non-HDL cholesterol (mmol/L)*^a^	*4.86 (4.45–5.45)*	*3.51 (3.14–3.78)*	*< 0.001*
*Fasting triglycerides (mmol/L)*	*1.7 ± 0.9*	*1.4 ± 0.7*	*0.007*
*Total fatty acids (µg/mL)*	*3868.8 ± 851.6*	*3122.6 ± 600.4*	*< 0.001*
Saturated fatty acids (%)	33.51 ± 2.56	32.95 ± 1.88	0.16
*Monounsaturated fatty acids (%)*	*26.56 ± 3.34*	*25.37 ± 2.46*	*0.025*
*Polyunsaturated fatty acids (%)*	*39.93 ± 4.61*	*41.65 ± 3.32*	*0.017*
*Fasting glucose (mmol/L)*^a^	*5.4 (4.8–5.8)*	*5.0 (4.8–5.5)*	*0.025*
*Glucose AUC OLTT (mmol/L min)*^a^	*9.72 (9.1–10.44)*	*9.43 (8.88–9.91)*	*0.046*
Glucose AUC OGTT (mmol/L min)^a^	3.68 (3.31–4.38)	3.31 (2.8–3.99)	0.018
Fasting insulin (µIU/mL)	16.0 ± 8.6	15.8 ± 8.2	0.95
HOMA-IR	3.7 ± 2.0	3.7 ± 2.2	0.78
Adiponectin (µg/mL)^a^	5.99 (4.38–8.72)	6.25 (3.84–9.55)	0.79
Leptin (ng/mL)a	34.74 (22.67–51.95)	29.16 (19.4–51.84)	0.29
*Fasting GIP (pg/mL)a*	*29.7 (19.3–44.7)*	*24.1 (15.9–33.6)*	*0.036*
*FGF19 (pg/mL)*	*147.9 ± 88.2*	*123.9 ± 90.6*	*0.035*
*FGF21 (pg/mL)*	*261.7 ± 160.6*	*214.2 ± 149.3*	*0.039*

Comparison between the high LDL-CH group and controls (unpaired t-test for normally or Mann–Whitney U-test for non-normally distributed ^a^ variables). Data shown as mean ± SD; otherwise (^a^) as median and interquartile range in parentheses except nominal variable (^b^) shown as % of subjects. ^b^ The chi-squared test was used for nominal variable. The statistically significant results are marked in italics

### The results of differential methylation analysis

**Fig. 1:**
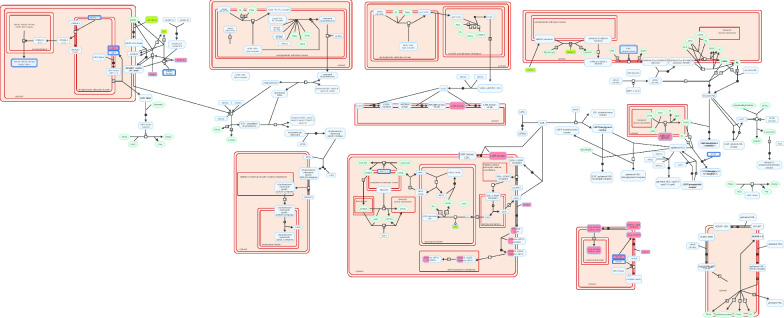
The results of genes methylation in plasma lipoprotein pathway based on the Reactome Pathway Browser. Genes coloured in green were hypomethylated while hypermethylated are in red. Only promoters are shown

In this preliminary study, we identified 7480 differentially methylated CpG sites, including 4905 CpG hypermethylated and 2575 hypomethylated sites. Data analysis in the Reactome Pathway Browser revealed engagement of 188 CpG probes, located in 143 genes in pathways related to lipid metabolism. The most interesting finding is the different methylation pattern of genes involved in: (1) lipoprotein assembly, remodeling and clearance (Fig. 1); (2) regulation of lipid metabolism by peroxisome proliferator-activated receptor alpha (PPARα); (3) regulation of cholesterol biosynthesis by sterol regulatory element binding protein (SREBP); (4) fatty acid metabolism and (5) triglyceride metabolism. In leukocytes of patients with elevated serum LDL-CH, we found hypomethylated promoters of the following genes involved in lipoprotein metabolism: *LPL, LIPA, ZDHHC8, PRKACA, AMN* and *FGF19*; hypermethylated promoters of *PCSK9, ABCG1, CLTC, AP2M1, AP2S1, LMF1, LSR, PRKACG, ANGPTL4, NR1H2,* and *PCSK5,* and hypermethylated inside the following genes: *LDLR, HDLBP, AP2A2* and *PCSK6* (Fig. 1 and Additional file 1: Table S1).

Enrichment analysis using BiNGO application showed possible involvement of genes in biological processes and identified probable molecular function. Results are presented in Table 3. Interestingly, the affected pathways in the group of obese with hypercholesterolemia included (1) lipoprotein remodeling in plasma (with hypomethylated promoter of *LPL* and hypermethylated *ABCG1* and *ABCA5*); (2) regulation of cholesterol storage (with hypomethylated promoters of *LPL* and *PPARG,* and hypermethylated *ABCG1* and *NR1H*2); and (3) cholesterol transport (with hypomethylated promoter of *LIPA* and hypermethylated *ABCA5, ABCG1* and *ABCG4*—responsible for cholesterol efflux) (Table 3a). Other genes differentially methylated were associated with (1) fatty acids biosynthesis (hypermethylated: *ELOVL3, ELOVL5,* and *ELOVL6,* and hypomethylated: *FASN, LPL, MLYCD* and *MCAT*); (2) fatty acid transport (hypermethylated: *ACSL6* and hypomethylated: *PPARD, PPARG, SLC25A20* and *SLC27A1*); and (3) fatty acid β-oxidation (hypermethylated: *ACADM* and *CPT1A* and hypomethylated: *AMN, CRAT* and *PPARD*). Some genes related to glucose homeostasis were also found to be hypermethylated: *FOXO3, NCOR2, TCF4* and *TCF7L2,* contrary to *PPARG* which was hypomethylated (Table 3a). The BiNGO plugin revealed probable molecular function of a set of genes as lipid binding, receptor binding, transporter activity and transcription factor binding (Table 3b).

**Table 3:** Results of statistically overrepresented biological processes and molecular function in set of differentially methylated genes

Description	p-value	Corr p-value	N	Hypermethylated	Hypomethylated
a. Gene Ontology_Biological Processes					
Lipid metabolic process	6.0610E−33	1.0849E−29	47	ABCG1 ACADM ACP6 ACSL6 AHRR CAV1 CPT1A ELOVL3 ELOVL5 ELOVL6 FDFT1 FDPS GPX1 GPX4 HDLBP NR1H2 LDLR LRP1 LRP5 PCSK9 PMVK RXRA SREBF1 SQLE	ABCB4 ACOT1 ACOT7 ACSF2 AGPAT2 AMN CRAT CYP1B1 CYP27B1 CYP51A1 DHCR7 FASN IL6ST INSIG1 LIPA LPL LRP8 MLYCD MCAT PPARD PPARG SLC27A1 STARD3
Regulation of lipid biosynthetic process	1.8237E−6	3.4005E−5	6	ABCG1 ACSL6 NR1H2	DHCR7 FGF19 SLC27A1
Negative regulation of lipid biosynthetic process	6.2381E−3	2.5581E−2	2		FGF19 SLC27A1
Fatty acid metabolic process	9.26E−17	2.37E−14	19	ACADM ACSL6 CPT1A ELOVL3 ELOVL5 ELOVL6 PPARA	ACOT1 ACOT7 ACSF2 AMN CRAT FASN LIPA LPL MCAT MLYCD PPARD SLC27A1
Cholesterol metabolic process	6.73E−19	6.02E−16	16	ABCG1 FDFT1 FDPS HDLBP LDLR PCSK9 PMVK RXRA SREBF1 SQLE	CYP51A1 DHCR7 LIPA PPARD SREBF2 STARD3
Triglyceride metabolic process	1.02E−08	4.81E−07	7	ACSL6 CAV1 CPT1A PCSK9	IL6ST LIPA LPL
Cholesterol biosynthetic process	5.57E−07	1.49E−05	5	FDFT1 FDPS PMVK	CYP51A1 DHCR7
Regulation of cholesterol storage	8.89E−09	4.42E−07	5	ABCG1 NR1H2 PPARA	LPL PPARG
Cholesterol transport	2.01E−07	6.09E−06	6	ABCA5 ABCG1 ABCG4 CAV1 LDLR	LIPA
Cholesterol efflux	1.00E−05	1.50E−04	4	ABCA5 ABCG1 ABCG4 CAV1	
Reverse cholesterol transport	5.57E−03	2.37E−02	2	ABCA5 ABCG1	
Regulation of cholesterol transport	4.57E−04	3.38E−03	3	NR1H2 LRP1	PPARG
Negative regulation of cholesterol storage	2.72E−08	1.08E−06	4	ABCG1 NR1H2 PPARA	PPARG
Fatty acid biosynthetic process	1.60E−06	3.12E−05	7	ELOVL3 ELOVL5 ELOVL6	FASN LPL MLYCD MCAT
Fatty acid transport	2.46E−08	1.05E−06	6	ACSL6 PPARA	PPARD PPARG SLC25A20 SLC27A1
Fatty acid beta-oxidation	1.22E−06	2.63E−05	5	ACADM CPT1A	AMN CRAT PPARD
Regulation of fatty acid oxidation	2.39E−06	4.32E−05	5	CPT1A PPARA	MLYCD PPARG SLC25A20
Long-chain fatty acid metabolic process	1.90E−03	1.04E−02	2	ACSL6	ACOT1
Regulation of macrophage derived foam cell differentiation	1.52E−08	6.63E−07	6	ABCA5 ABCG1 NR1H2 PPARA	LPL PPARG
Low-density lipoprotein receptor metabolic process	4.37E−05	5.00E−04	2	PCSK9	PPARG
Plasma lipoprotein particle remodeling	3.99E−04	3.11E−03	3	ABCA5 ABCG1	LPL
High-density lipoprotein particle remodeling	3.77E−03	1.75E−02	2	ABCA5 ABCG1	
Regulation of steroid metabolic process	3.09E−03	1.52E−02	3	ABCG1	FGF19 DHCR7
Glucose homeostasis	1.17E−06	2.58E−05	5	FOXO3 NCOR2 TCF4 TCF7L2	PPARG
Response to glucose stimulus	6.04E−05	6.47E−04	4	EP300 TCF7L2 TCF4	PPARD
Regulation of insulin secretion	4.82E−04	3.50E−03	3	CPT1A TCF4 TCF7L2	
Carbohydrate homeostasis	1.17E−06	2.58E−05	5	FOXO3 NCOR2 TCF4 TCF7L2	PPARG
Regulation of response to stress	5.02E−05	5.65E−04	10	AP2A2 AP2S1 AP2M1 CAV1 GPX4	FGF19 IL6ST IRAK1 PPARG PRKACA
Negative regulation of apoptosis	3.59E−03	1.70E−02	8	ANGPTL4 NKX2-6 SIN3A PCSK6 TCF4 TCF7L2	FGFR1 IRAK1
b. Gene Ontology_Molecular Function					
Lipid binding	2.19E−08	3.54E−06	15	ABCG1 AP2A2 AP2M1 CAV1 HDLBP PPARA RXRA S1PR4	ACOT7 ITPR1 LIPA LPL PPARD PPARG STARD3
Receptor binding	1.99E−06	1.16E−04	19	ANGPTL4 CAV1 EP300 LRP1 IL6ST IL13 NCOA2 NCOR2 NR1H2 PCSK9 RXRA TCF4 TCF7L2 TGFB1I1	CD8A FGF19 IRAK1 LPL PPARG
Transporter activity	1.44E−03	1.01E−02	16	ABCC1 ABCC9 ABCG1 AMN AP2A2 AP2S1 AP2M1 APC2 KCNJ6 LRP1 SLC27A1	ABCB4 FASN ITPR1 SLC25A20 STARD3
Transcription factor binding	1.26E−08	3.54E−06	17	CTCF GATA4 EP300 LRP1 MED21 NCOA2 NCOR2 NR1H2 RXRA SIN3A TCF4 TCF7L2 TGFB1I1	NFYC MED7 PPARD PPARG
Transcription cofactor activity	1.14E−05	4.22E−04	11	CTCF EP300 LRP1 MED21 NCOR2 NCOA2 RXRA SIN3A TGFB1I1	NFYC MED7
Lipoprotein binding	1.21E−03	9.26E−03	3	LDLR LRP1	LRP8

Analyses performed using BiNGO plugin in Cytoscape software. (a) Shows involvement of genes in biological processes and (b) in molecular function

In our study, hypermethylation of promoters of key genes regulating cholesterol metabolism such as *PCSK9, LRP1, ABCG1, ANGPTL4, SREBF1* and *NR1H2,* were found in obese patients with hypercholesterolemia. Furthermore, enhanced methylation of promoters of genes coding for transcription factors, such as *NFKB2, TCF4, GATA4, INSM1, CTCF, TCF7L2* and *SREBF1* and reduced methylation of *KLF14, PPARD* and *PPARG*, were found (Table 3a, b).

Locations of CpG sites in genes are presented in Additional file 1: Table S1.

## Discussion

This preliminary study showed different DNA methylation profiles in the leukocytes of obese, hypercholesterolemic patients (LDL-CH ≥ 3.4 mmol/L, n = 5) compared to the leukocytes methylome of obese subjects presenting serum LDL-CH levels < 3.4 mmol/L (n = 5). Main finding of this study is the identification of differentially methylated genes associated with lipid metabolism pathways. Among the lipid-related genes mostly significant were the following pathways: regulation of lipid metabolism by PPAR alpha; plasma lipoprotein assembly, remodeling, and clearance; metabolism of lipids; regulation of gene expression by SREBP, PPARA and NR1H2; LDL clearance and VLDLR internalisation and degradation (all located in the top ten of mostly significant pathways).

We have presented new loci of differential DNA methylation in genes that were previously found to be associated with dyslipidemia (*ABCG1, CPT1A, FASN, KLF14, LDLR, LPL, LRP1, PCSK9, PPARG* and *SREBF1*) (Mittelstraß and Waldenberger 2018; Pfeiffer et al. 2015; Rohde et al. 2019; Campanella et al. 2018) but also in new candidate genes, potentially related to hypercholesterolemia. Novel epigenetically regulated genes include *ABCG4, ANGPTL4, AP2A2, AP2M1, AP2S1, CLTC, FGF19, FGF1R, HDLBP, LIPA, LMF1, LRP5, LSR, NR1H2* and *ZDHHC8.* The unique set of differentially methylated genes were enriched in gene ontology to cholesterol and fatty acid metabolism, especially plasma lipoprotein formation and metabolism, reverse cholesterol transport, triglycerides degradation, fatty acids transport and β-oxidation.

Regarding lipoprotein assembly, remodeling and clearance, the reduced methylation in promoters of genes coding for *LPL* (Lipoprotein lipase), *LIPA* (Lipase A) and *ZDHHC8* (Zinc Finger DHHC-Type Containing 8) were found. Palmitoyl transferase ZDHHC8 mediates palmitoylation of ABCA1 and thus localization of this cholesterol transporter at the plasma membrane (Singaraja et al. 2009). Methylation of promoter of *LPL* gene in leukocytes and visceral adipose tissue was previously published in severe obese men (Guay et al. 2013). In this paper the association between *LPL* DNA methylation and *LPL* mRNA level in visceral adipose tissue and HDL cholesterol (HDL-CH) level was presented (Guay et al. 2013).

We detected hypomethylation in *FGF19* and *FGFR1* genes in leukocytes DNA of obese patients with hypercholesterolemia. The effect of FGF19 on TG and cholesterol levels may vary depending on the type of FGFR1 or FGFR4 receptor with which it interacts (Wu et al. 2013). Zhou et al. (2019) demonstrated that FGF19 promotes HDL biogenesis and cholesterol efflux from the liver, with increasing serum HDL and LDL cholesterol as a consequence.

The next set of genes found to be hypermethylated in our hypercholesterolemic patients: *PCSK9, CLTC, AP2A2*, *AP2M1* and *AP2S1*, are involved in the endocytosis of the ligand-bound LDL and VLDL receptors (Peterson et al. 2008; Mulkearns and Cooper 2012; Go and Mani 2012; Pearse et al. 2000). Although PCSK9 is principally expressed in the liver, PCSK9 gene promoter methylation is conserved across tissues and positively correlated with its expression (Lohoff et al. 2018). Recently it was found that FGF21 serves as a potential negative regulator of PCSK9 (Guo et al. 2016), which is in line with our observation that hypermethylation of *PCSK9* corresponds with higher circulating FGF21 levels in patients with hypercholesterolemia compared to control subjects.

Our study found differential methylation of the following genes encoding receptors involved in lipoprotein trafficking: hypermethylated *LDLR* (Low Density Lipoprotein Receptor), *LRP1* (LDL Receptor Related Protein 1), *LRP5* (LDL Receptor Related Protein 5), *LSR* (Lipolysis Stimulated Lipoprotein Receptor) and *NR1H2* (Nuclear Receptor Subfamily 1 Group H Member 2) and hypomethylated *LRP8* (LDL Receptor Related Protein 8). LRP1 together with LDLR play an essential role in binding and internalization of apoE- and apoB-containing lipoproteins regulating their cellular uptake (Dato and Chiabrando 2018). It has been found that DNA methylation of the *LRP1* gene (locus in 5′UTR) was previously detected in placental DNA and was correlated with maternal total cholesterol changes during pregnancy (Guay et al. 2020). In subsequent studies, a positive association between DNA methylation in *LRP1* gene and HDL-CH level was demonstrated in patients with metabolic syndrome (Castellano-Castillo et al. 2019). However, the exact location of the CpG sites has not been shown in these articles. We demonstrated hypermethylation of CpG sites located inside the fourth exon of the LDL receptor (*LDLR*) gene, however the consequences of methylation of that region have not yet been reported. Guay et al. (2020) presented correlation with DNA methylation in *LDLR* gene (CpG-A locus located between the fourth and fifth exon) with maternal total cholesterol level changes during pregnancy. Higher methylation degree of *LDLR* gene promoter in peripheral blood in atherosclerotic patients compared to healthy subjects was found (Zhi et al. 2007).

In presented study patients with elevated LDL-CH were characterized by hypermethylated CpG sites also within the promoters of the *ABCG1, ABCA5, ABCG4* and *CAV1* genes that are responsible for reverse cholesterol transport (Mauldin et al. 2008; Vaughan and Oram 2006). Pfeiffer et al. showed the association of CpG methylation in *ABCG1* (cg06500161, 21:43656587) with HDL cholesterol and triglycerides level in 1776 subjects in the KORA F4 cohort (Pfeiffer et al. 2015). Further studies demonstrated an association of methylation of (cg06500161) locus in *ABCG1* gene with its lower transcriptional activity, higher triglycerides level and higher triglycerides to HDL cholesterol ratio in 1941 obese individuals from four population-based European cohorts (Campanella et al. 2018). In our study we presented DNA hypermethylation in *ABCG1* gene in new CpG locus (21:42219751–42219800) in leukocytes of patients with high LDL-CH.

We also found altered DNA methylation in patients with hypercholesterolemia, in key genes associated with fatty acid transport and metabolism (hypomethylated: *PPARD, PPARG, SLC27A1*, *SLC27A3* and *SLC25A20*, and hypermethylated: *ANGPTL4, ACLS6, ACADM* and *CPT1A*)*.* These results indicate inhibited β-oxidation as the gene *CPT1A,* coding for the key enzyme in the carnitine-dependent transport and *ACADM* encoding acyl-CoA dehydrogenase medium chain catalysing the initial step of fatty acid β-oxidation, are hypermethylated (GeneCards—the human gene database www.genecards.org) (Stelzer et al. 2016). Frazier-Wood et al. showed association of methylation at 2 CpGs (cg00574958, 11:68607622; cg17058475, 11:68607737) in *CPT1A* gene with LDL and VLDL lipoprotein subfraction profile in CD4^+^ T cells (Frazier-Wood et al. 2014). They presented hypermethylation of 2 CpGs (cg00574958, cg17058475) in 5′UTR to be associated with decreased number of VLDL particles whereas one (cg00574958) was associated with a decrease in small, dense subfraction of LDL (Frazier-Wood et al. 2014). We presented DNA hypermethylation in new locus (11:68843060–68843119) located in the promoter of *CPT1A* gene in leukocytes of patients with high LDL-CH.

Interestingly, we identified differential methylation of genes coding for transcription factors important for lipid and glucose metabolism. The hypermethylated *SREBF1,* that regulates fatty acid and cholesterol metabolism and hypermethylated *TCF7L2,* important for glucose homeostasis, characterized hypercholesterolemic patients. Additionally the reduced methylation in transcription factors: *KLF14* gene*,* previously associated with metabolism of HDL-CH, adipocyte function, and *PPARD* and *PPARG*- receptors for fatty acids was found (Florath et al. 2016; Dekkers et al. 2016; Vitali et al. 2017; Argmann et al. 2017; Varga et al. 2011).

Despite the observed epigenetic changes in hypercholesterolaemia, we cannot state in our studies if the differential methylation is a consequence rather that a cause of high blood lipids. Based on the regulated pathways in our study as well as relevant literature (Pfeiffer et al. 2015; Dekkers et al. 2016; Rangel-Salazar et al. 2011) we suggest that differential methylation observed in epigenome wide association studies is likely an mixed picture of sites that are the cause and consequence of abnormal lipids levels. This is in line with previous studies where it was showed that blood lipids level influence DNA methylation (Dekkers et al. 2016). Studies on VLR (very low density—VLDL and LDL—rich lipoproteins mix) in human THP-1 macrophages showed that they induces global de novo methylation (Rangel-Salazar et al. 2011). Extension of these studies revealed that human native VLDL and LDL-rich lipoprotein mix, induces decrease in pro-inflammatory and cholesterol transport gene expression in consequence of DNA methylation. For instance *ABCG1* gene participating in cholesterol transport was down-regulated by VLR (Rangel-Salazar et al. 2011). Dekkers et al. analyzed genome wide DNA methylation in whole blood cells of 3296 individuals after Mendelian randomisation and demonstrated that higher LDL-CH induced higher methylation of a CpG (cg27168858) in *DHCR24* gene. Moreover higher TG levels induced lower methylation of 2 CpGs (cg00574958, cg17058475), which were associated with higher expression of *CPT1A.* High TG levels were also accompanied by hypermethylation of CpG (cg11024682), which was associated with lower expression of *SREBF1* gene. Additionally either lower TG or higher HDL-CH induced lower methylation of 2 CpGs (cg27243685, cg06500161), which was associated with higher expression of *ABCG1* gene (Dekkers et al. 2016). On the contrary, in article by Pfeiffer et al. (Pfeiffer et al. 2015) DNA methylation in *ABCG1*, *SREBF1* and *CPT1A* genes were presented rather as the cause not the consequence, of development of complex lipid-related diseases.

## Limitations

The main limitation of our study was the limited number of samples for genome-wide DNA methylation analysis. Thus results from the high-throughput method give rise for further studies on targeted genes methylation in all samples from the large cohort. We assessed DNA methylation in peripheral blood because it is easily accessible and its collection is acceptable by patients. Although epigenetic studies on different tissue samples are more informative, blood samples are generally used in most studies with non-surgical subjects. Nevertheless, previous studies demonstrated that hypermethylated CpG islands: *LEP, ADIPOQ* in adipose tissue or *PCSK9* in the liver overlap methylation status in the blood (Lohoff et al. 2018; Houde et al. 2015). Additionally Crujeiras et al. (2016) demonstrated that DNA methylation map in circulating leukocytes reflects subcutaneous adipose tissue methylation pattern. This suggests that DNA methylation analysis in leukocytes may reflect a methylation profile in other tissues (liver, adipose tissue or intestine) relevant for the pathogenesis of lipid and lipoprotein disorders. Furthermore various types of leukocytes (monocytes, neutrophils, mast cells and B and T lymphocytes) are associated with atherosclerosis suggesting that may actively response to hypercholesterolemia (Oguro 2019).

## Conclusions

In conclusion, our preliminary data implies epigenetic regulation of lipids profile, demonstrated as differential DNA methylation in leukocytes of obese individuals with elevated LDL cholesterol levels. Analysis of DNA methylation microarrays indicated that the most regulated processes are lipoprotein plasma clearance and metabolism, reverse cholesterol transport and cholesterol efflux and fatty acid uptake and β-oxidation. Analysis of DNA methylation status in peripheral blood could be a tool for identifying the pathognomonic processes related to the hypercholesterolemia and other obesity related complications. As DNA methylation is reversible and dependent on environmental factors, that gives the potential to influence the methylation status of lipids genes by the nutrition and healthy lifestyle to prevent the obesity-related complications.

## Supplementary information

**Additional file 1: Table S1. **Detail results of identified differentially methylated CpG sites.

## Data Availability

All data analysed during this study are included in this published article [and its additional information files].

## Abbreviations

AUC: Area under curves
BMI: Body mass index
CVD: Cardiovascular disease
FDR: False discovery rate
FGF19: Fibroblast growth factor 19
FGF21: Fibroblast growth factor 21
GIP: Glucose-dependent insulinotropic peptide
HDL: High density lipoprotein
HDL-CH: HDL cholesterol
HOMA-IR: Homeostasis Model Assessment Insulin Resistance
LDL: Low Density Lipoprotein
LDL-CH: LDL cholesterol
MBPs: Methyl-CpG binding proteins
MUFAs: Monounsaturated fatty acids
OGTT: Oral glucose tolerance test
OLTT: Oral lipid tolerance test
PUFAs: Polyunsaturated fatty acids
Q2: Median
(Q1; Q3): Interquartile range
SD: Standard deviation
TGs: Triglycerides
WHR: Waist-to-hip ratio
