# Supplementary material for: DNA methylation microarrays identify epigenetically regulated lipid related genes in obese patients with hypercholesterolemia
Source: Mol Med. 2020 Oct 7;26:93. doi: 10.1186/s10020-020-00220-z (PMC7539457; doi:10.1186/s10020-020-00220-z)
Supplement: Supplementary file 1 — Additional file 1: Table S1. Detail results of identified differentially methylated CpG sites. [file 10020_2020_220_MOESM1_ESM.docx]

Table S1

Detail results of identified differentially methylated CpG.

|  | **Gene symbol** | **Name** | **UniqueID** | **Genomic location of the methylated DNA fragmenth (according to hg38, GRCh38.p12)** | **adj.P.Val** | **p. Val** | **FC** | **Regulation** | **GeneBank Accession** | **Ensembl ID** | **EntrezID** | **MapLocation** |  |
| --- | --- | --- | --- | --- | --- | --- | --- | --- | --- | --- | --- | --- | --- |
| 1. 1 | | ABCA5 | ATP binding cassette subfamily A member 5 | A_17_P10435989 | 17:69326606-69326670 | 6.67E-06 | 1.15E-07 | 1.54 | hypermethylated | NM_018672 | ENSG00000154265 | 23461 | PROMOTER |
| 1. 2 | | ABCG1 | ATP binding cassette subfamily G member 1 | A_17_P17238714 | 21:42219751-42219800 | 7.09E-06 | 1.33E-07 | 1.64 | hypermethylated | NM_004915 | ENSG00000160179 | 9619 | PROMOTER |
| 1. 3 | | ABCG4 | ATP binding cassette subfamily G member 4 | A_17_P16508149 | 11:119149072-119149121 | 4.72E-06 | 2.04E-08 | 2.14 | hypermethylated | NM_001142505 | ENSG00000172350 | 64137 | PROMOTER |
| 1. 4 | | ACADM | acyl-CoA dehydrogenase medium chain | A_17_P00301957 | 1:75724472-75724522 | 4.59E-04 | 7.31E-05 | 1.36 | hypermethylated | NM_000016 | ENSG00000117054 | 34 | PROMOTER |
| 1. 5 | | ACSL6 | acyl-CoA synthetase long chain family member 6 | A_17_P04284026 | 5:132011126-132011175 | 3.64E-04 | 5.43E-05 | 1.96 | hypermethylated | NM_001009185 | ENSG00000164398 | 23305 | PROMOTER |
| 1. 6 | | AMN | amnion associated transmembrane protein | A_17_P16736764 | 14:102923590-102923639 | 2.86E-02 | 1.32E-02 | -1.36 | hypomethylated | NM_030943 | ENSG00000166126 | 81693 | PROMOTER |
| 1. 7 | | ANGPTL4 | angiopoietin like 4 | A_17_P10875679 | 19:8367619-8367668 | 1.20E-02 | 4.56E-03 | 1.34 | hypermethylated | NM_001039667 | ENSG00000167772 | 51129 | PROMOTER |
| 1. 8 | | AP2A2 | adaptor related protein complex 2 alpha 2 subunit | A_17_P27992042 | 11:1010659-1010708 | 1.36E-03 | 2.93E-04 | 1.99 | hypermethylated | NM_001242837 | ENSG00000183020 | 161 | INSIDE |
| 1. 9 | | AP2M1 | adaptor related protein complex 2 mu 1 subunit | A_17_P22964425 | 3:184175186-184175235 | 2.17E-05 | 1.11E-06 | 1.77 | hypermethylated | NM_001025205 | ENSG00000161203 | 1173 | PROMOTER |
| 1. 10 | | AP2S1 | adaptor related protein complex 2 sigma 1 subunit | A_17_P10978859 | 19:46860050-46860100 | 7.61E-05 | 6.82E-06 | 2.69 | hypermethylated | NM_001301076 | ENSG00000042753 | 1175 | PROMOTER |
| 1. 11 | | AP2S1 | adaptor related protein complex 2 sigma 1 subunit | A_17_P31615029 | 19:46860581-46860630 | 8.72E-04 | 1.66E-04 | 1.56 | hypermethylated | NM_001301076 | ENSG00000042753 | 1175 | PROMOTER |
| 1. 12 | | CAV1 | caveolin 1 | A_17_P25974151 | 7:116525561-116525618 | 3.47E-04 | 5.11E-05 | 1.63 | hypermethylated | NM_001172895 | ENSG00000105974 | 857 | PROMOTER/ CTCF |
| 1. 13 | | CLTC | clathrin heavy chain | A_17_P10398043 | 17:59618894-59618958 | 2.61E-03 | 6.72E-04 | 1.88 | hypermethylated | NM_001288653 | ENSG00000141367 | 1213 | PROMOTER |
| 1. 14 | | CLTC | clathrin heavy chain | A_17_P17000968 | 17:59619787-59619837 | 3.46E-03 | 9.57E-04 | 1.54 | hypermethylated | NM_001288653 | ENSG00000141367 | 1213 | PROMOTER |
| 1. 15 | | CPT1A | carnitine palmitoyltransferase 1A | A_17_P07855391 | 11:68843060-68843119 | 6.50E-03 | 2.11E-03 | 1.87 | hypermethylated | NM_001031847 | ENSG00000110090 | 1374 | PROMOTER |
| 1. 16 | | CRAT | carnitine O-acetyltransferase | A_17_P27322499 | 9:129110788-129110837 | 2.68E-03 | 6.94E-04 | -1.34 | hypomethylated | NM_000755 | ENSG00000095321 | 1384 | DIVERGENT_PROMOTER |
| 1. 17 | | CTCF | CCCTC-binding factor | A_17_P30625068 | 16:67564235-67564299 | 8.13E-03 | 2.79E-03 | 1.73 | hypermethylated | NM_001191022 | ENSG00000102974 | 10664 | PROMOTER |
| 1. 18 | | ELOVL3 | ELOVL fatty acid elongase 3 | A_17_P07437523 | 10:102226041-102226090 | 1.21E-05 | 4.17E-07 | 1.43 | hypermethylated | NM_152310 | ENSG00000119915 | 83401 | PROMOTER/ CTCF |
| 1. 19 | | ELOVL5 | ELOVL fatty acid elongase 5 | A_17_P04743741 | 6:53349149-53349204 | 3.19E-04 | 4.58E-05 | 1.62 | hypermethylated | NM_001242828 | ENSG00000012660 | 60481 | PROMOTER |
| 1. 21 | | ELOVL6 | ELOVL fatty acid elongase 6 | A_17_P03345552 | 4:110197975-110198039 | 2.79E-03 | 7.29E-04 | 1.58 | hypermethylated | NM_001130721 | ENSG00000170522 | 79071 | PROMOTER |
| 1. 22 | | ELOVL6 | ELOVL fatty acid elongase 6 | A_17_P03345548 | 4:110197624-110197673 | 2.25E-02 | 9.85E-03 | 1.76 | hypermethylated | NM_001130721 | ENSG00000170522 | 79071 | PROMOTER |
| 1. 23 | | FASN | fatty acid synthase | A_17_P17028210 | 17:82100878-82100926 | 3.71E-04 | 5.57E-05 | 1.72 | hypermethylated | NM_004104 | ENSG00000169710 | 2194 | PROMOTER |
| 1. 24 | | FASN | fatty acid synthase | A_17_P10495141 | 17:82100931-82100980 | 5.92E-04 | 1.01E-04 | 1.68 | hypermethylated | NM_004104 | ENSG00000169710 | 2194 | PROMOTER |
| 1. 25 | | FGF19 | fibroblast growth factor 19 | A_17_P07858926 | 11:69703463-69703512 | 1.19E-03 | 2.47E-04 | -1.55 | hypomethylated | NM_005117 | ENSG00000162344 | 9965 | PROMOTER |
| 1. 26 | | FGFR1 | fibroblast growth factor receptor 1 | A_17_P16087944 | 8:38467971-38468020 | 1.16E-05 | 3.87E-07 | -1.44 | hypomethylated | NM_001174063 | ENSG00000077782 | 2260 | PROMOTER |
| 1. 27 | | FOXO3 | forkhead box O3 | A_17_P04972480 | 6:108559133-108559187 | 5.36E-06 | 5.47E-08 | 1.72 | hypermethylated | NM_001455 | ENSG00000118689 | 2309 | PROMOTER |
| 1. 29 | | GATA4 | GATA binding protein 4 | A_17_P05958586 | 8:11702890-11702939 | 1.37E-03 | 2.96E-04 | 1.61 | hypermethylated | NM_001308093 | ENSG00000136574 | 2626 | PROMOTER |
| 1. 30 | | GATA4 | GATA binding protein 4 | A_17_P26237912 | 8:11708120-11708184 | 1.81E-02 | 7.55E-03 | 2.04 | hypermethylated | NM_001308093 | ENSG00000136574 | 2626 | PROMOTER |
| 1. 32 | | HDLBP | high density lipoprotein binding protein | A_17_P02006246 | 2:241240098-241240151 | 6.24E-04 | 1.08E-04 | 1.77 | hypermethylated | NM_001243900 | ENSG00000115677 | 3069 | INSIDE |
| 1. 33 | | INSM1 | INSM transcriptional repressor 1 | A_17_P31748781 | 20:20366845-20366896 | 2.79E-05 | 1.62E-06 | 2.14 | hypermethylated | NM_002196 | ENSG00000173404 | 3642 | PROMOTER |
| 1. 34 | | KLF14 | Kruppel like factor 14 | A_17_P26040333 | 7:130733683-130733731 | 9.05E-06 | 2.34E-07 | -1.49 | hypomethylated | NM_138693 | ENSG00000266265 | 136259 | PROMOTER/CTCF |
| 1. 35 | | LDLR | low density lipoprotein receptor | A_17_P10883462 | 19:11105553-11105597 | 7.66E-06 | 1.59E-07 | 1.58 | hypermethylated | NM_000527 | ENSG00000130164 | 3949 | INSIDE |
| 1. 36 | | LIPA | lipase A, lysosomal acid type | A_17_P16371202 | 10:89251643-89251692 | 2.91E-02 | 1.35E-02 | -1.45 | hypomethylated | NM_000235 | ENSG00000107798 | 3988 | PROMOTER |
| 1. 37 | | LMF1 | lipase maturation factor 1 | A_17_P09884182 | 16:964975-965035 | 9.82E-06 | 2.73E-07 | 2.36 | hypermethylated | NM_001352017 | ENSG00000103227 | 64788 | PROMOTER |
| 1. 38 | | LPL | lipoprotein lipase | A_17_P16076101 | 8:19939501-19939550 | 8.64E-06 | 2.13E-07 | -1.70 | hypomethylated | NM_000237 | ENSG00000175445 | 4023 | PROMOTER |
| 1. 39 | | LRP1 | LDL receptor related protein 1 | A_17_P08375976 | 12:57128097-57128148 | 1.46E-02 | 5.78E-03 | 1.43 | hypermethylated | NM_002332 | ENSG00000123384 | 4035 | PROMOTER/CTCF |
| 1. 40 | | LRP5 | LDL receptor related protein 5 | A_17_P07853290 | 11:68348109-68348158 | 7.40E-06 | 1.47E-07 | 1.62 | hypermethylated | NM_001291902 | ENSG00000162337 | 4041 | INSIDE |
| 1. 41 | | LRP5 | LDL receptor related protein 5 | A_17_P07853704 | 11:68449076-68449133 | 2.16E-04 | 2.76E-05 | 2.72 | hypermethylated | NM_001291902 | ENSG00000162337 | 4041 | INSIDE |
| 1. 42 | | LRP5 | LDL receptor related protein 5 | A_17_P07853539 | 11:68413738-68413794 | 9.17E-04 | 1.77E-04 | 2.02 | hypermethylated | NM_001291902 | ENSG00000162337 | 4041 | INSIDE |
| 1. 43 | | LRP8 | LDL receptor related protein 8 | A_17_P00203415 | 1:53326514-53326563 | 3.86E-03 | 1.10E-03 | -1.30 | hypomethylated | NM_001018054 | ENSG00000157193 | 7804 | PROMOTER |
| 1. 44 | | LSR | lipolysis stimulated lipoprotein receptor | A_17_P10947000 | 19:35249327-35249377 | 2.69E-03 | 6.96E-04 | 1.82 | hypermethylated | NM_001260489 | ENSG00000105699 | 51599 | PROMOTER |
| 1. 45 | | MCAT | malonyl-CoA-acyl carrier protein transacylase | A_17_P17288443 | 22:43143145-43143194 | 1.14E-05 | 3.73E-07 | -1.73 | hypomethylated | NM_014507 | ENSG00000100294 | 27349 | PROMOTER |
| 1. 46 | | MLYCD | malonyl-CoA decarboxylase | A_17_P16919698 | 16:83898938-83898987 | 3.94E-02 | 5.53E-04 | 2.37 | hypomethylated | NM_012213 | ENSG00000103150 | 23417 | PROMOTER |
| 1. 47 | | NCOR2 | nuclear receptor corepressor 2 | A_17_P08667315 | 12:124506367-124506416 | 4.12E-05 | 2.87E-06 | 1.63 | hypermethylated | NM_001077261 | ENSG00000196498 | 9612 | PROMOTER |
| 1. 48 | | NCOR2 | nuclear receptor corepressor 2 | A_17_P08666454 | 12:124336537-124336601 | 1.37E-04 | 1.51E-05 | 1.56 | hypermethylated | NM_001077261 | ENSG00000196498 | 9612 | INSIDE |
| 1. 49 | | NCOR2 | nuclear receptor corepressor 2 | A_17_P29103379 | 12:124324776-124324825 | 1.51E-03 | 3.36E-04 | 2.31 | hypermethylated | NM_001077261 | ENSG00000196498 | 9612 | INSIDE |
| 1. 50 | | NCOR2 | nuclear receptor corepressor 2 | A_17_P08666935 | 12:124426781-124426830 | 2.44E-03 | 6.17E-04 | 2.02 | hypermethylated | NM_001077261 | ENSG00000196498 | 9612 | PROMOTER FLANK REGION |
| 1. 52 | | NR1H2 | nuclear receptor subfamily 1 group H member 2 | A_17_P17153894 | 19:50376246-50376295 | 2.17E-03 | 5.29E-04 | 1.60 | hypermethylated | NM_001256647 | ENSG00000131408 | 7376 | PROMOTER |
| 1. 53 | | PCSK5 | proprotein convertase subtilisin/kexin type 5 | A_17_P06747064 | 9:75891814-75891865 | 8.40E-03 | 2.91E-03 | 1.61 | hypermethylated | NM_001190482 | ENSG00000099139 | 5125 | PROMOTER |
| 1. 54 | | PCSK6 | proprotein convertase subtilisin/kexin type 6 | A_17_P09878486 | 15:101398382-101398437 | 4.14E-04 | 6.41E-05 | 1.70 | hypermethylated | NM_001291309 | ENSG00000140479 | 5046 | INSIDE |
| 1. 55 | | PCSK9 | proprotein convertase subtilisin/kexin type 9 | A_17_P00210461 | 1:55040339-55040388 | 3.20E-05 | 1.99E-06 | 1.91 | hypermethylated | NM_174936 | ENSG00000169174 | 255738 | PROMOTER |
| 1. 56 | | PPARA | peroxisome proliferator activated receptor alpha | A_17_P11530800 | 22:46214774-46214824 | 8.54E-05 | 7.99E-06 | 1.59 | hypermethylated | NM_001001928 | ENSG00000186951 | 5465 | INSIDE |
| 1. 57 | | PPARA | peroxisome proliferator activated receptor alpha | A_17_P11530798 | 22:46214592-46214641 | 1.54E-05 | 6.35E-07 | 2.54 | hypermethylated | NM_001001928 | ENSG00000186951 | 5465 | INSIDE |
| 1. 58 | | PPARD | peroxisome proliferator activated receptor delta | A_17_P15805159 | 6:35342814-35342863 | 5.37E-05 | 4.21E-06 | 2.77 | hypomethylated | NM_001171818 | ENSG00000112033 | 5467 | PROMOTER |
| 1. 59 | | PPARG | peroxisome proliferator activated receptor gamma | A_17_P15413233 | 3:12288274-12288323 | 4.08E-03 | 1.18E-03 | -1.57 | hypomethylated | NM_001330615 | ENSG00000132170 | 5468 | PROMOTER |
| 1. 60 | | PRKACA | protein kinase cAMP-activated catalytic subunit alpha | A_17_P17111828 | 19:14118313-14118362 | 8.21E-05 | 5.02E-04 | -1.69 | hypomethylated | NM_001304349 | ENSG00000072062 | 5566 | PROMOTER |
| 1. 61 | | PRKACG | protein kinase cAMP-activated catalytic subunit gamma | A_17_P06716070 | 19:1445460-1445509 | 4.72E-06 | 4.91E-09 | 2.33 | hypermethylated | NM_002732 | ENSG00000165059 | 5568 | PROMOTER/ CTCF |
| 1. 63 | | RXRA | retinoid X receptor alpha | A_17_P06999054 | 9:134439717-134439768 | 7.34E-04 | 1.33E-04 | 1.77 | hypermethylated | NM_001291920 | ENSG00000186350 | 6256 | INSIDE/CTCF |
| 1. 64 | | RXRA | retinoid X receptor alpha | A_17_P16273032 | 9:134408916-134408965 | 1.43E-02 | 5.62E-03 | 1.29 | hypermethylated | NM_001291920 | ENSG00000186350 | 6256 | INSIDE |
| 1. 65 | | SLC25A20 | solute carrier family 25 member 20 | A_17_P02224440 | 3:48898546-48898596 | 1.78E-03 | 4.12E-04 | -1.58 | hypomethylated | NM_000387 | ENSG00000178537 | 788 | PROMOTER |
| 1. 66 | | SLC27A1 | solute carrier family 27 member 1 | A_17_P17115752 | 19:17470497-17470546 | 5.37E-03 | 1.66E-03 | -1.58 | hypomethylated | NM_198580 | ENSG00000130304 | 376497 | PROMOTER |
| 1. 68 | | SREBF1 | sterol regulatory element binding transcription factor 1 | A_17_P10256746 | 17:17824804-17824855 | 1.55E-03 | 3.47E-04 | 1.79 | hypermethylated | NM_001005291 | ENSG00000072310 | 6720 | PROMOTER |
| 1. 69 | | SREBF2 | sterol regulatory element binding transcription factor 2 | A_17_P32200174 | 22:41910687-41910736 | 6.03E-04 | 1.04E-04 | -1.52 | hypomethylated | NM_004599 | ENSG00000198911 | 6721 | DOWNSTREAM |
| 1. 70 | | TCF4 | transcription factor 4 | A_17_P10726944 | 18:55590080-55590144 | 6.83E-03 | 2.25E-03 | 2.39 | hypermethylated | NM_001083962 | ENSG00000196628 | 6925 | PROMOTER |
| 1. 71 | | TCF4 | transcription factor 4 | A_17_P31326733 | 18:55321779-55321843 | 1.15E-02 | 4.29E-03 | 1.50 | hypermethylated | NM_001083962 | ENSG00000196628 | 6925 | PROMOTER |
| 1. 72 | | TCF4 | transcription factor 4 | A_17_P10726932 | 18:55588542-55588606 | 8.49E-03 | 1.99E-02 | 1.93 | hypermethylated | NM_001146274 | ENSG00000148737 | 6934 | PROMOTER |
| 1. 73 | | TCF7L2 | transcription factor 7 like 2 | A_17_P07488729 | 10:112950872-112950936 | 2.58E-04 | 1.23E-03 | 1.38 | hypermethylated | NM_001146274 | ENSG00000148737 | 6934 | PROMOTER |
| 1. 74 | | TCF7L2 | transcription factor 7 like 2 | A_17_P27879309 | 10:112953104-112953168 | 8.51E-03 | 2.95E-03 | 3.50 | hypermethylated | NM_001146274 | ENSG00000148737 | 6934 | PROMOTER |
| 1. 75 | | ZDHHC8 | zinc finger DHHC-type containing 8 | A_17_P17256157 | 22:20131969-20132018 | 5.95E-03 | 1.89E-03 | -1.59 | hypomethylated | NM_001185024 | ENSG00000099904 | 29801 | PROMOTER |

Only probes described in the manuscript are shown.
